# High-frequency home self-collection of capillary blood correlates *IFI27* expression kinetics with SARS-CoV-2 viral clearance

**DOI:** 10.1172/JCI173715

**Published:** 2023-12-01

**Authors:** Fang Yun Lim, Soo-Young Kim, Karisma N. Kulkarni, Rachel L. Blazevic, Louise E. Kimball, Hannah G. Lea, Amanda J. Haack, Maia S. Gower, Terry Stevens-Ayers, Lea M. Starita, Michael Boeckh, Ollivier Hyrien, Joshua T. Schiffer, Ashleigh B. Theberge, Alpana Waghmare

**Affiliations:** 1 Vaccine and Infectious Disease Division, Fred Hutchinson Cancer Center, Seattle, Washington, USA.; 2 Department of Chemistry,; 3 Brotman Baty Institute,; 4 Department of Genome Sciences,; 5 Department of Medicine,; 6 Department of Urology, and; 7 Department of Pediatrics, University of Washington, Seattle, Washington, USA.; 8 Seattle Children’s Research Institute, Seattle, Washington, USA.

**Keywords:** COVID-19, Infectious disease, Innate immunity

**To the Editor:** Blood transcriptional profiling is a powerful tool to evaluate immune responses to infection; however, blood collection via traditional phlebotomy remains a barrier to precise characterization of the immune response in dynamic infections, especially when early and frequent measurements are desired. During acute viral infections, the complex interplay between innate and acquired immunity drives a rapidly evolving immune landscape; a delicate balance between pro- and antiinflammatory molecules can pivot an individual toward effective pathogen elimination or excessive host tissue damage. Thus, tracking the immune kinetics at deep temporal resolution during this period can offer valuable mechanistic insights into pathogenesis of the infection. Our team recently developed a home collection toolkit (*home*RNA) that enables self-collection of liquid capillary blood coupled with immediate in-field stabilization of RNA (1). This toolkit uniquely enables frequent longitudinal measurements from nonhospitalized individuals presenting with mild-to-moderate disease during the most contagious and dynamic stage of their infection. During the COVID-19 pandemic, we coupled *home*RNA with self-collected nasal swabs to profile the kinetics of host and viral factors during mild-to-moderate SARS-CoV-2 infections.

Previously vaccinated and unvaccinated COVID-19^+^ (*n =* 39) and uninfected (*n =* 5) participants self-collected blood using *home*RNA every other day, in addition to completing daily nasal swabs and symptom surveys across a 2-week observational window (Supplemental Figure 1, A–D). The demographics of all participants are summarized in Supplemental Table 1. High-resolution temporal kinetics of 773 genes spanning 56 immune-associated pathways were profiled across 232 longitudinal blood samples (Supplemental Figure 1C) using digital counting of native mRNA (nCounter) (Supplemental Figure 1D). We fitted 3 generalized additive mixed models (GAMMs), using smoothed functions of time (days after symptom onset) to describe the temporal dynamics of gene expression and examine their potential association with disease and vaccination status (Supplemental Methods and Supplemental Table 2). In parallel, we modeled the temporal trends of coregulated gene networks using time-course gene set analyses (TcGSA) to evaluate pathway-level dynamics across disease and vaccination subgroups. Uninfected participants (all female) showed remarkably stable immune kinetics, with no dynamic genes or pathways identified in both GAMM and TcGSA analyses (Figure 1A), demonstrating the reproducibility and stability of expression signatures over time from participant-collected samples. In contrast, previously unvaccinated COVID-19^+^ participants showed broad perturbations in the periphery, with 60% of genes (*n =* 470; adjusted *P* < 0.1) and 74% of pathways (*n =* 211; adjusted *P* < 0.05) showing statistically significant temporal dynamicity (Figure 1A). Over 62% of gene dynamicity observed during acute COVID-19 infection was driven by responses in unvaccinated individuals (Supplemental Figure 2A) and was notable for heightened expression of interferon-stimulated genes (ISGs) (Figure 1B). Previously vaccinated COVID-19^+^ individuals demonstrated a muted transcriptional response in both gene and pathway-level analyses (Figure 1A) and fewer dynamic ISG responses during the observational window (Figure 1B). Dynamic genes shared between previously vaccinated and unvaccinated COVID-19^+^ participants were enriched in innate antiviral responses (Supplemental Figure 2B), while those specific to unvaccinated individuals were enriched in cytokine signaling/production and leukocyte adhesions/migration (Supplemental Figure 2C). Despite being limited to a small cohort and late disease observation period, we were able to observe robust infection and vaccination-associated signatures from frequent intraindividual measurements.

Next, we interrogated blood transcriptional correlates of upper airway viral load (VL) using temporally aligned blood and swab samples to better understand mechanisms that potentially drive viral clearance. A single ISG, *IFI27*, showed strong positive correlation (Pearson’s *r* = 0.73) with VL for all COVID-19^+^ participants (Figure 1C). Moderate correlations were noted for other ISGs (*IFI44*, *OAS1*, *OAS2*, *OAS3*, *RSAD2*, *MX1*), the cytidine deaminase (*APOBEC3G*) involved in host-dependent viral editing, chemokine receptors (*CCR5*, *CX3CR1*), DNA/RNA sensor (*ZBP1*), the transcription factor (*XBP1*) associated with endoplasmic reticulum stress, and cytolytic granule protein perforin-1 (*PRF1*) (Figure 1D). Prior studies demonstrated evidence of APOBEC-mediated host-editing of SARS-CoV-2 (2). However, the role of *APOBEG3G* in both host-editing of SARS-CoV-2 genome and pathogenesis of SARS-CoV-2 remains unclear (3), despite its known role in restricting HIV replication (4). Although *IFI27* is known to be upregulated during viral infections (5), the correlation between *IFI27* and VL temporal trajectories has not been established prior to this study. Here, we demonstrated that *IFI27* expression in the periphery has strong temporal alignment with VL trajectories in individual participants, regardless of vaccination status (Figure 1E), suggesting a potential role of this gene in mediating viral clearance. In sum, we present the first application to our knowledge of a home self-blood collection system for profiling host immune kinetics during acute-phase SARS-CoV-2. The capability of this tool to generate reproducible longitudinal immune signatures and capture dynamic or transient host responses extends its utility to mechanistic studies requiring frequent longitudinal outpatient sampling, including a broad array of disease states beyond COVID-19.

## Supplementary Material

Supplemental data

Supporting data values

## Figures and Tables

**Figure 1:**
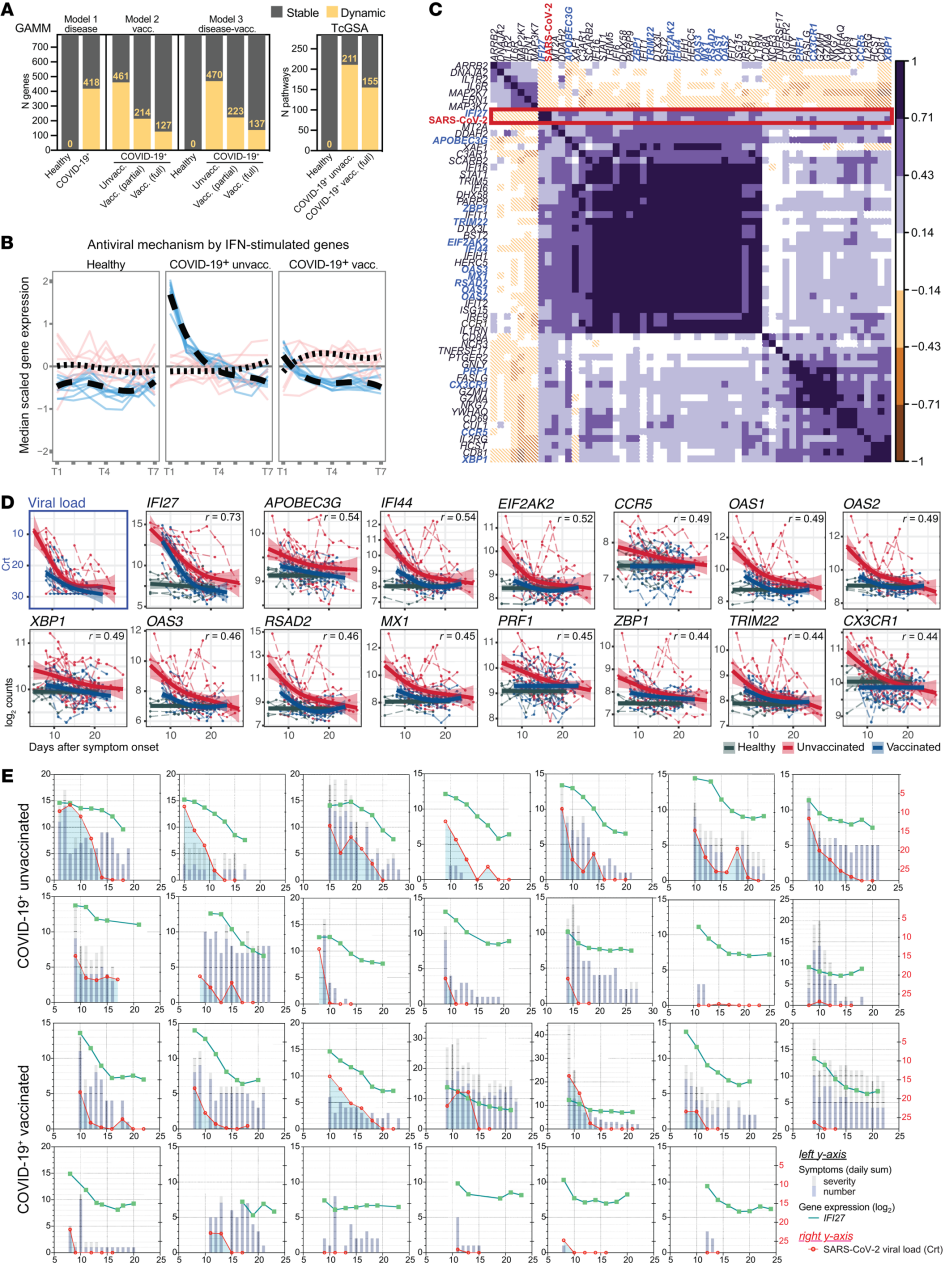
Self-blood collection captures temporal alignment between *IFI27* and SARS-CoV-2 VL. (**A**) Dynamic genes and pathways identified through GAMM and TcGSA, respectively. (**B**) Spaghetti plots depicting time trends of a dynamic antiviral pathway identified through TcGSA. Colored solid lines represent the scaled median expression of one gene across all participants, and black dotted lines represent the smoothed median of all genes for a given time trend. Colors denote genes in distinct time trend clusters (blue, cluster 1; pink, cluster 2). (**C**) Pearson’s correlation matrix of genes with ≥0.3 absolute correlation coefficient with SARS-CoV-2 VL in temporally aligned blood and swab samples. (**D**) Temporal kinetics of SARS-CoV-2 VL and genes showing moderate-to-strong associations with VL in previously vaccinated and unvaccinated COVID-19^+^ participants. Solid lines represent generalized additive model smoothing across all participants within a group, and colored shades represent the 95% confidence interval. Green, red, and blue colors denote healthy, COVID-19^+^ unvaccinated, and COVID-19^+^ vaccinated participants, respectively. Dotted lines represent individual temporal trajectories of gene. (**E**) Temporal kinetics of *IFI27* gene expression, VL, and symptom burden in individual participants.

## Version 1 12/01/2023

Electronic publication
