# Supplementary material for: High-frequency home self-collection of capillary blood correlates IFI27 expression kinetics with SARS-CoV-2 viral clearance
Source: J Clin Invest. 2023 Dec 1;133(23):e173715. doi: 10.1172/JCI173715 (PMC10688975; doi:10.1172/JCI173715)
Supplement: Supplemental data [file jci-133-173715-s154.pdf]

High-frequency home self-collection of capillary blood correlates *IFI27* expression kinetics to SARS-CoV-2 viral clearance

Fang Yun Lim[1,2], Soo-Young Kim[1], Karisma N. Kulkarni[1], Rachel L. Blazevic[1], Louise E. Kimball[1], Hannah G. Lea[2], Amanda J. Haack[2], Maia. S. Gower[2], Terry Stevens-Ayers[1], Lea M. Starita[3,4], Michael Boeckh[1,5], Ollivier Hyrien[1], Joshua T. Schiffer[1,5]*, Ashleigh B. Theberge[2,6]*, Alpana Waghmare[1,7,8]*.

[1]Vaccine and Infectious Disease Division, Fred Hutchinson Cancer Center; Seattle Washington, U.S.A.

[2]Department of Chemistry, University of Washington; Seattle, Washington, U.S.A.

[3]Brotman Baty Institute, University of Washington, Seattle

[4]Department of Genome Sciences, University of Washington, Seattle

[5]Department of Medicine, University of Washington; Seattle, Washington, U.S.A.

[6]Department of Urology, University of Washington; Seattle, Washington, U.S.A.

[7]Department of Pediatrics, University of Washington; Seattle, Washington, U.S.A.

[8]Seattle Children's Research Institute; Seattle, Washington, U.S.A.

*co-senior authors contribute equally

Correspondence: Alpana Waghmare, 1100 Fairview Ave N. Seattle, WA 98109, U.S.A. +1(206)667-7329; (awaghmar@fredhutch.org)

Ashleigh B. Theberge, Department of Chemistry, Box 351700 Seattle, WA 98195, U.S.A. (abt1@uw.edu)

Joshua T. Schiffer, 1100 Fairview Ave N. Seattle, WA 98109, U.S.A. +1-206-667-7359; (jschiffe@fredhutch.org)

COI: All financial support reported below are outside of the submitted work. AW has clinical trial support from Pfizer, Ansun Biopharma, Allovir, GlaxoSmithKline, and Vir Biotechnology, grants from Amazon, and receives personal fees from Vir Biotechnology. MB has clinical research support from Ansun Biopharma, Amazon, GSK, Vir Biotechnology, and Merck and receives personal fees from Allovir, Moderna, and Merck. JTS has clinical trial support from Aicuris and receives personal fees from Glaxo Smith Kline and Pfizer. ABT has ownership in Stacks to the Future, LLC, and research support from Ionis Pharmaceuticals.

DETAILED METHODS

Study Design and Participant Characteristics.

The objective of this study was to test the application of a home-use blood sampling and RNA stabilization kit (*home*RNA) to capture transcriptomic signatures of disease and track their evolution over time in an out-of-clinic (remote) unsupervised use setting. We conducted a longitudinal observational case-control study in a cohort of individuals with acute COVID-19 infection (n = 39) and healthy uninfected controls (n = 5) (**Fig. S1A**). Healthy uninfected controls were recruited from the general population. For the control group, healthy adults with no history of respiratory symptoms or SARS-CoV-2 positivity within 14 days of eligibility screen were recruited from the general population. In the COVID-19+ group, both vaccinated and unvaccinated COVID-19+ adult participants with a positive SARS-CoV-2 nucleic acid amplification test within 7 days of eligibility screen were recruited through the COVID-19 Clinical Research Center. Participants who completed the full COVID-19 vaccination series at least two weeks prior to study enrollment were classified as vaccinated. Participants who received at least a single dose of COVID-19 vaccination but did not meet the two-weeks requirement above were classified as partially vaccinated. Participants who did not receive any doses of COVID-19 vaccination prior to or during their participant were classified as unvaccinated. Each study participant collected blood samples using *home*RNA every other day (7 sampling timepoints), collected daily nasal swab samples (14 sampling timepoints) and completed daily symptom surveys (14 surveys) over a two-week period to track blood transcriptional response, VL kinetics, and symptom progression respectively. This study was conducted at the Fred Hutchinson

Cancer Center, Seattle WA. All sample collections were performed remotely by study participants. Participants were recruited to the study between January – September 2021.

home**RNA blood collection**

Detailed characterization of the *home*RNA kit usage has been previously described (1). Briefly, participants were instructed to use the Tasso-SST blood collection device on the upper arm. General *home*RNA blood collection procedure included i) warming the collection site using a heat pack to facilitate blood flow, ii) cleaning the site with provided alcohol wipes, iii) adhering the Tasso-SST blood collection device to the cleaned area, iv) pressing a button to activate the lancet and initiate blood collection, v) removing the Tasso-SST device after five minutes or when the collection tube is full (whichever comes first), and vi) covering the puncture site with provided sterile bandage. Immediately after blood collection, participants were instructed to disconnect the Tasso-SST blood tube and reconnect it to the stabilizer tube to initiate mixing of the collected blood and stabilizer. The stabilized blood samples were transferred into a 50-mL conical tube containing a custom-design tube insert and mailed back to the University of Washington at ambient temperature using overnight courier services directly to a secure -20ºC freezer. Returned blood samples were transferred to a -80ºC freezer within 1-2 days post-receipt for storage until ready for RNA extraction.

home**RNA blood sampling kit assembly and device fabrication.**

Detailed description of the *home*RNA kit and device design has been previously described (1). Briefly, the Tasso-SST blood collection device was purchased from Tasso, Inc. The RNA stabilizer reagent vial, adaptor, and cap were injection molded out of polycarbonate (PC: Makrolon 2407) by Protolabs, Inc (Maple Plain, MN). Prior to fabrication, all components of the stabilizer tube were sonicated in 70% ethanol (v/v) for 30 minutes and air dried. The adaptor was bonded onto the reagent vial using Dymax MDÒ UV-curable medical grade glue (#1450-M-UR-SC). Bonded parts were UV-cured for 12 minutes at 30ºC using a Form Cure UV resin-curing chamber (Formlabs). The fabricated stabilizer vial was filled with 1.3 mL of RNA-stabilizing solution (RNAlater^{TM}),

capped, and packaged in an impulse-sealed Tyvek pouch. Each *home*RNA blood collection kit was assembled with a Tasso-SST blood collection device, a stabilizer tube, instructions for use, and all other kit components required for the participant to perform blood collection.

Respiratory specimen collection and viral load (VL) kinetics

COVID-19+ participants were asked to collect anterior nasal swab samples from both nostrils using a sterile polyurethane custom foam swab (Puritan Ref# 251805PFSC2ARROW). Respiratory swab specimens were stored dry (without universal transport medium), mailed back to the lab, and immediately transferred to -80ºC storage until ready for pathogen analysis. To quantify VL, total nucleic acid was extracted using Magna Pure 96 small total nucleic acid isolation kit (Roche Diagnostics). Isolated nucleic acid was screened for the presence of SARS-CoV-2 and multiple other respiratory viral and bacterial pathogens (**Fig. S1D)** by TaqMan-based quantitative reverse transcription polymerase chain reaction (RT-qPCR) on the OpenArray platform (Thermo Fisher). Based on high concordance between Crt and Ct values reported by the manufacturer, TaqMan relative threshold values (Crt) were used to estimate VL. A pathogen was classified as detected in a respiratory specimen when its Crt value was ≤ 28. To track VL kinetics, respiratory specimens with time-matched *home*RNA blood samples were serially assayed until samples were negative for any respiratory pathogen in two consecutive samples.

SARS-CoV-2 sequencing

Sequencing was attempted for a single SARS-CoV-2 positive respiratory sample with the lowest Crt value (highest VL) from each COVID-19+ participant. Nucleic acid was extracted using the Magna Pure 96 small total nucleic acid isolation kit (Roche Diagnostics) and sequencing libraries prepared using the COVIDSeq kit (Illumina). Artic V4 primers were used (https://community.artic.network/t/sars-cov-2-version-4-scheme-release/312). Viral genomes were sequenced using the NextSeq2000 P200 kit (Illumina), and the SARS-CoV-2 reference genome (Wuhan/Hu-1/2019; Genbank accession MN908947) was used to assemble consensus genomes

using a modified iVar pipeline. Nextstrain augur software was used to align viral sequences and construct phylogenetic trees for variant determination.

Participant reported outcomes and *home*RNA device use surveys

Daily symptom surveys were administered virtually via REDCap (2) in the COVID-19 case group. A total of 26 respiratory and non-respiratory symptom categories were presented in the survey. Participants scored each symptom category based on severity (0 = none, 1 = mild, 2 = moderate, 3 = severe). COVID-19 vaccination manufacturer and dates were obtained to further classify COVID-19 cases into vaccinated and unvaccinated subgroups. Participants also completed a device use survey after each *home*RNA blood collection to assess device usability, kit integrity during transport, blood collection parameters, and pain levels experienced during *home*RNA blood collection.

Gene expression analysis

RNA isolation, cleanup, and concentration. All procedures using commercial kits were performed according to the manufacturer's recommended protocol unless otherwise noted. For PAXgene venipuncture samples, total RNA was extracted using the PAXgene Blood RNA kit. For *home*RNA-stabilized blood samples, total RNA was isolated using the Ribopure™ Blood RNA Isolation Kit. RNA concentrations were measured using the Take3 microvolume plates on the BioTek Cytation 5 multimode reader (Agilent technologies). RNA quality was measured on the Bioanalyzer 2100 (Agilent Technologies) using the RNA 6000 Nano kit (Agilent Technologies #5067-1511) for samples with concentrations ≥ 5 ng/µL. RNA samples with concentration < 5 ng/µL, RIN values were measured using the RNA 6000 Pico kit (Agilent Technologies #5067-1513). For nCounter gene expression analysis, RNA samples were column purified and concentrated using the Monarch RNA Cleanup Kit (NEB #T2030). Samples were stored at -80ºC until ready for gene expression analysis.

nCounter data quality control and normalization. nCounter RCC files of all samples were pre-processed on the nSolver^{TM} software (nanoString) to obtain normalized expression counts. Quality control (QC) measures were applied to all participant samples. Imaging QC was assessed by measuring the percent field of view (FOV) successfully scanned within each sample. Samples with scanned FOV < 75% were flagged for removal. Binding density QC was assessed by measuring the density of the reporter probe on the cartridge surface within each sample. Samples with binding density outside of 0.1 - 2.25 spots/square micron were flagged for removal. The correlations between the observed counts for all six positive ERCC control probes (Positives A – F) and their spike-in synthetic nucleic acids (0.125 fM – 128 fM) were used to determine a sample's positive control linearity. Samples with positive control linearity of < 0.95 were flagged for removal. Gene expression count normalization was performed on all samples that passed the QC metrics above. For normalization, all samples were subjected to i) positive control normalization, ii) codeset content normalization, and iii) panel standard normalization. Selection of reference genes was performed using ROSALIND® (https://rosalind.bio/) based on nanoString recommendations. Nine reference genes (*ABCF1, GUSB, HRPT1, MRPS7, NMT1, NRDE2, PGK1, SDHA, TBP*) with stable expression within the dataset as determined by geNorm were used in codeset content normalization.

nCounter analysis. For gene expression analysis, direct detection and digital counting of native RNA transcripts was performed on the nCounter Pro Analysis System (nanoString). For each participant sample, 50-100 ng of total RNA samples were hybridized to the nCounter Host Response Panel codeset (nanoString) containing both capture and molecular barcoded-reporter probes to target genes. 773 genes associated with the host response to infectious diseases and 12 candidate reference (housekeeping) genes were targeted. Target-probe hybrids were immobilized on a cartridge, aligned, and digitally counted on the nCounter Pro digital analyzer. Two Host Response codeset versions (Host Response v1.0 and Host Response v1.1) were used to generate the dataset. A panel standard containing identical counts of all target panel genes was run alongside participant samples for each of the two codeset versions to normalize counts between the two codeset.

Statistics

Generalized additive mixed model (GAMM) analysis. We used GAMM to explore associations between gene expression and COVID-19 disease status over time, adjusting for age, sex, nCounter Host Response codeset versions (v1.0 and v1.1), and vaccination status (3). All fitted GAMMs included subject-specific random intercepts to account for potential intra-subject correlation and described longitudinal gene expression data using smoothed functions of time, defined as the number of days since first positive PCR test or onset of symptoms for COVID-19 positive participants. For healthy uninfected participants, an initial time point (Day 1) was defined as the median initial time point for COVID-19+ participants. We developed three GAMMs: i) *Model 1* included COVID-19 disease status as factor; ii) *Model 2* adjusted for vaccination but not COVID-19 disease status, and was fitted to data from COVID-19+ participants only; iii) *Model 3* was similar to *Model 1*, except that it adjusted for vaccination and COVID-19 disease status using a 4-level categorical variable with levels defined as (1) (COVID-19 negative, fully vaccinated), (2) (COVID-19 positive, unvaccinated), (3) (COVID-19 positive, partially vaccinated), and (4) (COVID-19 positive, fully vaccinated). This allows us to distinguish disease-dependent response in both previously vaccinated and unvaccinated individuals. P-values were adjusted for multiple comparisons by controlling the false discovery rate (FDR) using the Benjamini-Hochberg (BH) procedure (4). Adjusted p-values < 0.1 were used to identify differentially expressed transcripts. GAMM analyses were carried out using the gamm4 R package (5), setting the number of bases set to 5 in all analyses (5). Gene ontology (GO) enrichment analysis was performed using the ClusterProfiler R package (6) in R Statistical Software (v4.2.1, R Core Team 2022) (7).

Time-course geneset analysis (TcGSA). Dynamic pathways between healthy, COVID-19+ unvaccinated, and COVID-19+ vaccinated participant groups were identified using the TcGSA package available on CRAN (http://cran.r-project.org/web/packages/TcGSA/index.html) (8). The TcGSA analysis utilized mixed models to compute the likelihood ratios for predefined genesets and identify gene trends that are not stable over time (8). To account for the heteroskedasticity of the nCounter gene expression data, regularized log (rlog) transformation

of normalized counts was performed in DESeq2 (http://bioconductor.org/packages/DEseq2/) prior to TcGSA analysis (9). Reactome pathway database was used to query dynamic genesets within our expression dataset. To ensure samples from individual participants are represented within each analyzed time-course group, sampling timepoints were used as the time variable in the analysis. Dynamic pathways between three participant groups (COVID-19+ unvaccinated, COVID-19+ previously vaccinated, and healthy uninfected controls) were analyzed adjusting for age, sex, and nCounter codeset version using a linear time function. P-values were adjusted for multiple comparisons by controlling the FDR using the Benjamini-Yekatueli (BY) procedure. Adjusted p-values < 0.05 were used to identify significant genesets. ISG pathway (R-HSA-1169410) were visualized using the rlog expression values for each gene within the queried pathway and aligned to sampling timepoints 1 – 7 (T1-T7) and displayed as spaghetti plots with each solid colored line representing the median scaled gene expression for a given gene across all participants within that group and each black dotted line represents the smoothed median of all genes for a given time trend. Temporal heterogeneity (distinct time trend clusters) within the Reactome pathways was identified by the gap statistics (capped at a maximum of three clusters).

Study Approval. The study was approved by the Fred Hutchinson Institutional Review Board [protocol approval number: FH10523]. All study participants provided informed consent prior to participation in the study.

Data availability. All data are available in the supporting data file. Gene expression dataset can be found in GSE243348. All statistical analyses were conducted using R. Packages used to perform analyses are specified in the statistical section. Scripts developed to perform analyses are available from the corresponding author upon request.

SUPPLEMENTAL FIGURES

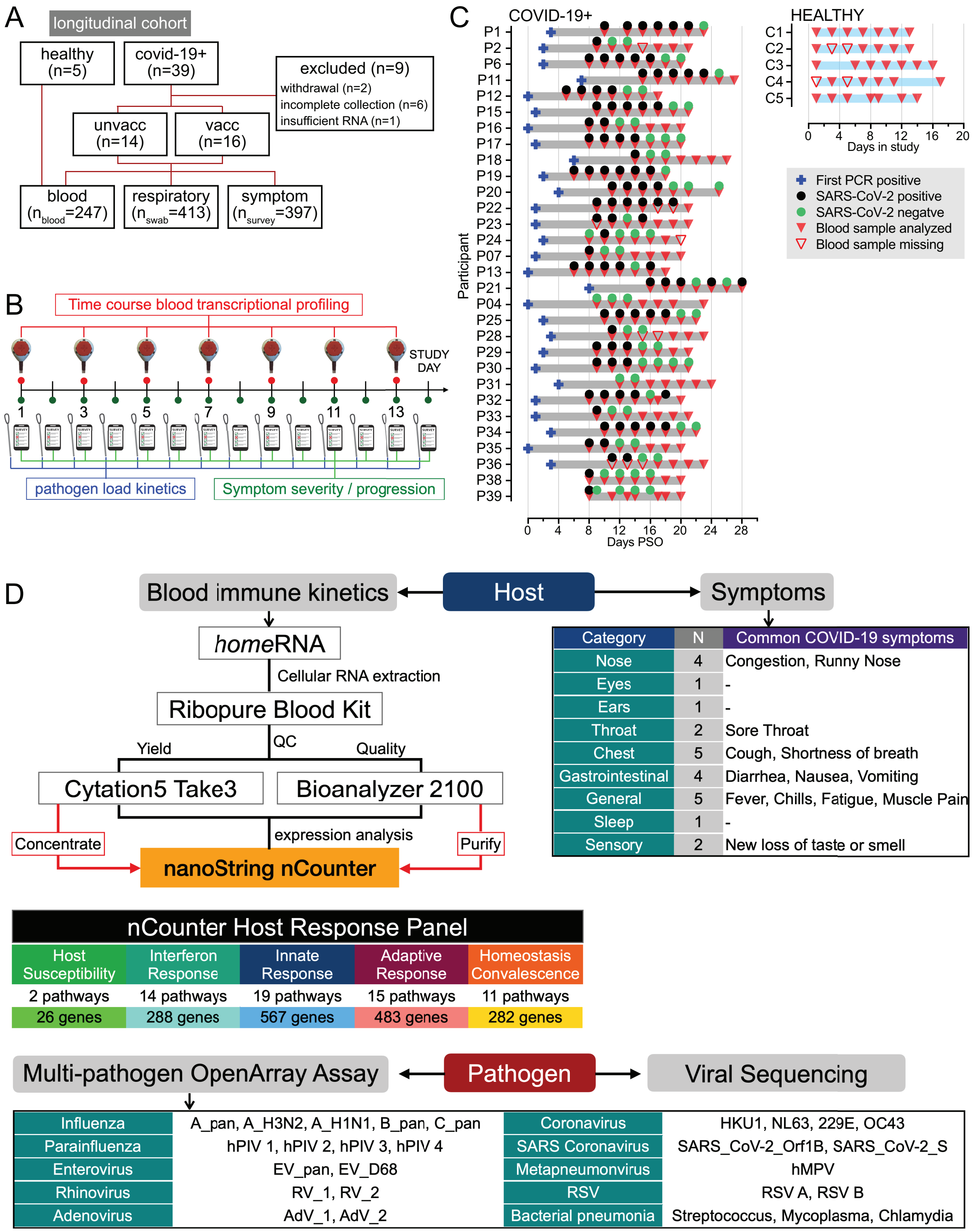

Figure S1. Longitudinal transcriptional profiling of the SARS-CoV-2 acute phase response. A) Flow chart of cohort characteristics. **B**) Study design depicting frequency of blood and nasal swab collection and symptom

burden assessment. **C**) Disease timeline and participant blood and nasal swab samples aligned to days post symptom onset (PSO) (Day 0) in both COVID-19+ participants and healthy uninfected participants. Blue cross denotes first PCR positive day; black and green circles denote SARS-CoV-2 positive and negative nasal swab samples respectively; solid red triangles denote blood samples used in gene expression analysis while transparent red triangles denote missing blood samples. **D**) Flowchart depicting both host- and pathogen-associated outcomes measured in the study and their respective sample analysis workflows.

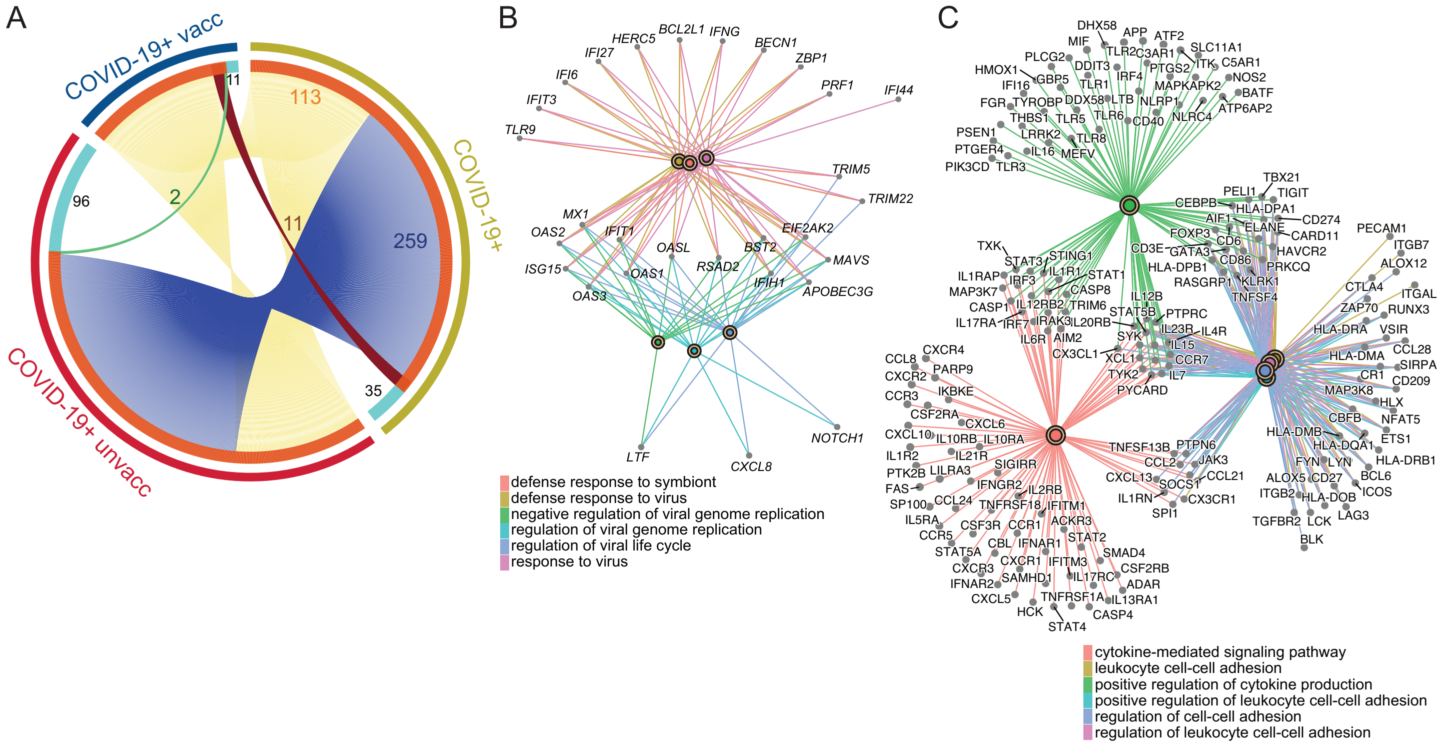

Figure S2. **Gene ontology enrichment analysis of dynamic genes identified from GAMM. A)** Circos plot showing overlap of dynamic genes between disease and vaccination groups. Colored lines link identical genes between each group. The inner circle represents gene lists, where hits are arranged along the arc. Dark orange denotes genes that hit multiple lists; blue denotes genes unique to a particular list. Gene-concept network plots depicting linkages between genes and biological pathways (concepts) for the top 6 GO biological process pathways enriched in the **B**) significantly dynamic genes shared across all three COVID-19+, COVID-19+ unvaccinated, and COVID-19 vaccinated participant grous (n=113) and **C**) dynamic genes unique to the COVID-19+ unvaccinated response (n=355). Each biological pathway is represented by a different colored node (circle) and its pathway genes are linked by the same solid colored lines. Genes are clustered based on their pathway associations. Genes with multiple associations are clustered away from those associated with a single pathway.

SUPPLEMENTAL TABLES

Table 1. Demographics and Clinical Characteristics of Study Participants

	Longitudinal cohort (n = 35)		
	COVID-19+ (n = 30)		Healthy (n = 5)
	unvaccinated (n = 14)	vaccinated (n = 16)	
**Demographics**			
**Sex,** N (% cohort)			
Female	9 (25.7%)	10 (28.6%)	5 (14.3%)
Male	5 (14.3%)	6 (17.1%)	0 (0%)
**Age (years),** Median (IQR)	31 (29 - 41.5)	36.5 (31.25 - 52.5)	42 (39.5 - 54.5)
**Ethnicity,** N (% cohort)			
Hispanic	3 (8.6%)	1 (2.9%)	0 (0%)
Non-Hispanic	11 (31.4%)	15 (42.9%)	5 (14.3%)
**Race,** N (% cohort)			
Asian	1 (2.9%)	0 (0%)	1 (2.9%)
Black/African American	0 (0%)	0 (0%)	1 (2.9%)
White	8 (22.9%)	15 (42.9%)	3 (8.6%)
Two or more races	3 (8.6%)	1 (2.9%)	0 (0%)
Unknown	2 (5.7%)	0 (0%)	0 (0%)
**Clinical characteristics**			
**Weight (lbs)**, Median (IQR)	165 (146.8 - 193.5)	179 (146.3 - 201.3)	160 (142.5 - 184.5)
**Height (inches),** Median (IQR)	67.5 (66 - 69.9)	67.5 (64.3 - 70)	67 (59 - 71)
**Disease timeline (days),** Median (IQR) [min-max]			
post 1st symptom onset	9 (8 - 10.25) [5 - 15]	9 (8 - 10.75) [6 - 16]	NA
post 1st SARS-CoV-2+ test	7 (6 - 8) [4 - 8]	8 (7 - 8) [6 - 9]	NA

Table S2. Parameters of fitted GAMM models

GAMM	Covariates	Vaccination Level	Samples analyzed	Contrast groups	Smoothed functions[1]
Model 1	age<br>sex<br>codeset<br>disease		all participants	covid19:healthy	s(days):healthy<br>s(days):covid19
Model 2	age<br>sex<br>codeset<br>vaccination	Unvaccinated<br>Vaccinated (Partial)<br>Vaccinated (Full)	covid-19+ participants only	covid19 vacc:unvacc<br>covid19 partial:unvacc	s(days):unvacc<br>s(days):vacc(partial)<br>s(days):vacc(full)
Model 3	age<br>sex<br>codeset<br>vaccination	Unvaccinated<br>Vaccinated (Partial)<br>Vaccinated (Full)<br>Healthy	all participants	covid19 unvacc:healthy<br>covid19 vacc:healthy<br>covid19 partial:healthy	s(days):healthy<br>s(days):unvacc<br>s(days):vacc(partial)<br>s(days):vacc(full)

[1]smoothed functions of time were modeled for each disease and vaccination status using the number of days since symptom onset [s(days)].

ACKNOWLEDGEMENTS

Funding:

- National Institutes of Health grant R01AI153087 (AW)
- R35GM128648 (ABT, for in-lab developments of *home*RNA)
- Fred Hutchinson Cancer Center COVID-19 Pilot grant (AW)
- Packard Research Fellowship from the David and Lucile Packard Foundation (ABT).

Author contributions:

We would like to thank Drs. Damien Chaussabel, Boris Hejblum, and Darawan Rinchai for project discussions. We would also like to thank the study participants for their participation in this research.

Conceptualization: FYL, ABT, AW

Methodology: FYL, SYK, AJH, OH, ABT, AW

Investigation: FYL, SYK, LMS, OH, ABT, AW

Visualization: FYL, SYK, HGL

Funding acquisition: ABT, AW

Project administration: KK, RLB, HGL, MSG

Supervision: LMS, MB, OH, ABT, AW

Writing – original draft: FYL, ABT, AW

Writing – review & editing: FYL, SYK, LMS, MB, JTS, OH, ABT, AW

References and Notes

1. Haack AJ, et al. homeRNA: A Self-Sampling Kit for the Collection of Peripheral Blood and Stabilization of RNA. *Anal Chem.* 2021;93(39):13196-203.
2. Harris PA, et al. Research electronic data capture (REDCap)--a metadata-driven methodology and workflow process for providing translational research informatics support. *J Biomed Inform.* 2009;42(2):377-81.
3. Lin X, and Zhang D. Inference in Generalized Additive Mixed Models by Using Smoothing Splines. *Journal of the Royal Statistical Society Series B (Statistical Methodology).* 1999;61(2):381-400.
4. Benjamini Y, and Hochberg Y. Controlling the False Discovery Rate: A Practical and Powerful Approach to Multiple Testing. *Journal of the Royal Statistical Society: Series B (Methodological).* 1995;57(1):289-300.
5. Wood S, and Scheipl F. 2017.
6. Yu G, et al. clusterProfiler: an R package for comparing biological themes among gene clusters. *OMICS.* 2012;16(5):284-7.
7. R Development Core Team. Vienna, Austria: R Foundation for Statistical Computing; 2022.
8. Hejblum BP, et al. Time-Course Gene Set Analysis for Longitudinal Gene Expression Data. *PLoS Comput Biol.* 2015;11(6):e1004310.
9. Love MI, et al. Moderated estimation of fold change and dispersion for RNA-seq data with DESeq2. *Genome Biol.* 2014;15(12):550.
